# LC-MS/MS Quantitation of HILIC-Enriched *N*-glycopeptides Derived from Low-Abundance Serum Glycoproteins in Patients with Narcolepsy Type 1

**DOI:** 10.3390/biom13111589

**Published:** 2023-10-28

**Authors:** Mojgan Atashi, Cristian D. Gutierrez Reyes, Vishal Sandilya, Waziha Purba, Parisa Ahmadi, Md. Abdul Hakim, Firas Kobeissy, Giuseppe Plazzi, Monica Moresco, Bartolo Lanuzza, Raffaele Ferri, Yehia Mechref

**Affiliations:** 1 Department of Chemistry and Biochemistry, Texas Tech University, Lubbock, TX 79409, USA; mojgan.atashi@ttu.edu (M.A.); cristian.d.gutierrez-reyes@ttu.edu (C.D.G.R.); vishal.sandilya@ttu.edu (V.S.); wpurba@ttu.edu (W.P.); pahmadi@ttu.edu (P.A.); md-abdul.hakim@ttu.edu (M.A.H.); 2 Department of biochemistry and molecular genetics, Faculty of Biochemistry and Molecular Genetics, American University of Beirut, Beirut 11072020, Lebanon; fkobaissy@msm.edu; 3 Department of Neurobiology, Center for Neurotrauma, Multiomics & Biomarkers (CNMB), Neuroscience Institute, Morehouse School of Medicine, Atlanta, GE 30310, USA; 4 IRCCS, Instituto delle Scienze Neurologiche di Bologna, 40124 Bologna, Italy; giuseppe.plazzi@unibo.it (G.P.); monica.moresco@ausl.bologna.it (M.M.); 5 Department of Biomedical, Metabolic and Neural Sciences, University of Modena and Reggio Emilia, 41125 Modena, Italy; 6 Sleep Research Center, Department of Neurology IC, Oasi Research Institute-IRCCS, 94018 Tronia, Italy; blanuzza@oasi.en.it (B.L.); rferri@oasi.en.it (R.F.)

**Keywords:** NT1, HILIC-enriched *N*-glycopeptides, biomarker glycoproteins

## Abstract

Glycoproteomic analysis is always challenging because of low abundance and complex site-specific heterogeneity. Glycoproteins are involved in various biological processes such as cell signaling, adhesion, and cell–cell communication and may serve as potential biomarkers when analyzing different diseases. Here, we investigate glycoproteins in narcolepsy type 1 (NT1) disease, a form of narcolepsy characterized by cataplexy—the sudden onset of muscle paralysis that is typically triggered by intense emotions. In this study, 27 human blood serum samples were analyzed, 16 from NT1 patients and 11 from healthy individuals serving as controls. We quantified hydrophilic interaction liquid chromatography (HILIC)-enriched glycopeptides from low-abundance serum samples of controls and NT1 patients via LC-MS/MS. Twenty-eight unique *N*-glycopeptides showed significant changes between the two studied groups. The sialylated *N*-glycopeptide structures LPTQNITFQTESSVAEQEAEFQSPK HexNAc_6_, Hex_3_, Neu5Ac_2_ (derived from the ITIH4 protein) and the structure IVLDPSGSMNIYLVLDGSDSIGASNFTGAK HexNAc_5_, Hex_4_, Fuc_1_ (derived from the CFB protein), with *p* values of 0.008 and 0.01, respectively, were elevated in NT1 samples compared with controls. In addition, the *N*-glycopeptide protein sources Ceruloplasmin, Complement factor B, and ITH4 were observed to play an important role in the complement activation and acute-phase response signaling pathways. This may explain the possible association between the biomarkers and pathophysiological effects.

## 1. Introduction

Narcolepsy is a neurological sleep disorder characterized by a pentad of symptoms: excessive daytime sleepiness, cataplexy, hypnagogic and hypnopompic hallucinations, sleep paralysis (especially on awakening), and disturbed nocturnal sleep [1]. The prevalence of narcolepsy with cataplexy ranges between 25 and 50 per 100,000 individuals [2,3]. The two primary symptoms of narcolepsy type 1 (NT1) are excessive daytime sleepiness and cataplexy, which is a sudden loss of muscle tone while awake, usually triggered by powerful, typically positive emotions. Along with sleep paralysis and nighttime sleep disturbances, narcoleptic patients may also experience hypnagogic or hypnopompic hallucinations, which are hallucinations that occur as a person is falling unconscious or waking up [4,5]. A lack of hypocretin/orexin signaling in NT1 in the posterior part of the hypothalamus [6] may arise from an autoimmune process. Although this is speculative, DNA and epidemiological evidence support the idea. According to epidemiological evidence, narcolepsy may be brought on by influenza A, hemolytic streptococcal illnesses, and diseases of the immune system; the existence of an autoimmune process is still debated [7]. Narcolepsy without cataplexy and/or evidence of orexin deficiency is called narcolepsy type 2. Although narcolepsy can affect anyone at any age, it is widely accepted that symptoms usually appear during childhood adolescence, with some cases having an onset during young adulthood. There are no issues based on sex because narcolepsy affects both men and women equally [2].

Glycoproteins are essential for many important biological functions [8,9]. Glycosylation is one of the most prevalent post-translational modifications, accounting for up to 50% of the human proteome. Glycoproteins take part in different biological processes such as protein folding, cell formation, cell–cell contact and adhesion, immune defense, fertilization, viral propagation, parasitic infection, dissolution of blood clots, inflammation, and cancer metastasis [10,11,12]. Glycoproteomics represents the glycosylation position that takes place in addition to the composition of glycoproteins. Therefore, the microheterogeneity of each glycosylation site can be assessed using glycoproteomics. The two different kinds of glycosylation are N-linked and O-linked [13]. During N-linked glycosylation, an asparagine (N) group is chemically joined to a glycoform. The peptide backbone sequence has the amino acid sequence NXS/T, where X is not a proline. The five-saccharide center of N-linked glycans, which is made up of two *N*-acetylglucosamine (GlcNAc) and three mannose (Man) units, has two antennae accessible for additional glycosylation. In contrast, when an amino acid with a hydroxyl functional group, such as a serine (S) or threonine (T), is attached to a glycoform without a particular sequence structure, O-linked glycans are produced. O-linked glycans can produce up to eight distinct core structures rather than just one [11]. Saccharides can be attached to the core positions [14].

In order to identify and quantify protein glycosylation, liquid chromatography coupled to mass spectrometry (LC/MS and LC/MS/MS) is currently the technique most commonly used [15]. Numerous studies have demonstrated that both LC/MS and LC-MS/MS methods can be used to effectively identify glycoproteins [16,17,18]. Due to the micro- and macro-heterogeneity of glycoproteins, the research of glycopeptides is challenging [19]. LC-MS/MS is one of the most applicable methods for glycoconjugate analysis, offering various fragmentation mechanisms and valuable structural data [20,21,22]. The comparable fragmentation patterns and structural glycan isomers of glycopeptides, such as those of sialic acid linkages, are difficult to differentiate from isomeric glycopeptides. Therefore, it is essential to distinguish between isomeric structures prior to conducting an MS study. RPLC (reverse-phase liquid chromatography), HILIC (hydrophilic interaction liquid chromatography), and PGC-LC (porous graphitized carbon liquid chromatography) are some techniques for separating isomeric glycopeptides [23,24,25,26,27,28,29,30,31]. These liquid chromatography techniques can be coupled with tandem MS for accurate analysis of isomeric glycopeptides.

Utilizing the polarity of the glycan, hydrophilic interaction liquid chromatography (HILIC) selectively enriches glycopeptides. The cellulose material attracts the polar glycopeptides to a larger extent than it does the tryptic peptides [14].After tryptic digestion, glycopeptides are significantly less common, whereas proteins are more prevalent. Glycopeptides also have a low ionization efficiency when subjected to MS analysis. The challenges of glycopeptide analysis are made more difficult by these two findings [32,33]. Enriching glycopeptide samples prior to MS analysis has therefore become a prevalent practice [14]. Enrichment permits the selective binding of the glycopeptides by removing the majority of other interfering species (such as tryptic peptides). Among the frequently used enrichment methods are immunoprecipitation [34,35], hydrazide enrichment [36,37], and lectin affinity chromatography (LAC) [38,39]. Hydrophilic interaction liquid chromatography (HILIC) significantly enriches glycopeptides with the change in the polarity of the glycan; the cellulose material binds the polar glycopeptides more firmly than the tryptic peptides [40,41]. Here, we study glycoproteins in NT1, a life-altering disease for which there is currently no cure. Therefore, it is crucial to identify biomarkers to improve the diagnosis of narcolepsy type 1. In this study, we depleted 27 blood serum samples to derive low abundance proteins and for the first time utilized HILIC enrichment following LC-MS/MS analysis to enhance *N*-glycopeptide levels in narcolepsy samples. The utilization of this method facilitated the identification of potentially valuable biomarker glycopeptides, enabling effective differentiation between samples from individuals with NT1 and those who are healthy.

## 2. Materials and Methods

### 2.1. Clinical Information of NT1 Patients and Healthy Controls

A total of 16 patients clinically diagnosed with narcolepsy type 1 (NT1) and 11 healthy people participated in this study. All participants agreed to be the subject of this study and provided their written consent. Approval from the local ethics committee was obtained prior to conducting this study. Serum samples from patients with NT1 were collected from the Department of Neurology and Sleep Medicine, Oasi Research Institute (IRCCS), and the Department of Neurology, University of Bologna, Italy, between April and June 2018. *The International Classification of Sleep Disorders (3rd ed.)* presents a clinical diagnosis of NT1 and four criteria were recommended for a laboratory to follow to record these patient diagnoses: (a) unequivocal cataplexy during laboratory testing; (b) persistent daytime sleepiness; (c) at least two SOREMPs and an 8 min mean sleep latency during the multiple sleep latency tests; and (d) evidence of cerebrospinal fluid orexin A deficiency, when available [20]. Since the human leukocyte antigen (HLA) DQB1 0602 allele is closely associated with narcolepsy, the patients’ lab results were based on the presence of the mentioned antigen and any other medical conditions were excluded. More details and information are provided in Table 1. “Controls” in the results refers to healthy participants. None of the patients had a medical history of diabetes, hypertension, or any neurological conditions other than NT1, and they were not taking any medications when blood samples were collected. 

### 2.2. Chemicals and Reagents

Ammonium bicarbonate (ABC), dithiothreitol (DTT), and iodoacetamide (IAA) were purchased from Sigma-Aldrich (St. Louis, MO, USA). HPLC-grade water, acetonitrile (ACN), and mass-spectrometry-grade formic acid (FA) were bought from Fisher Scientific (Fair Lawn, NJ, USA). Mass-spectrometry-grade trypsin/Lys-C was acquired from Promega (Madison, WI, USA). Depletion buffers (Buffer A and Buffer B), 0.22 µm spin filters, and 5K MWCO spin concentrators were purchased from Agilent Technologies (Santa Clara, CA, USA). Spectrometric-grade trifluoroacetic acid (TFA) 99% was obtained from Aldrich Chemical Co., Inc. (Milwaukee, WI, USA). The Micro BCA Protein Assay Kit was purchased from Thermo Fisher Scientific Inc. (Rockford, IL, USA).

### 2.3. Depletion of High-Abundance Proteins in Serum Samples

This study mainly focused on the differentially expressed low-abundance glycoproteins in disease and control samples. To obtain the low-abundance glycoproteins, an affinity-based column named the Agilent Human Affinity Removal System Column (4.6 × 100 mm) was employed. This column can remove 14 high-abundance proteins albumin, IgG, antitrypsin, IgA, transferrin, haptoglobin, fibrinogen, alpha-2-macroglobulin, alpha-1-acid glycoprotein, IgM, apolipoprotein AI, apolipoprotein AII, complement C3, and transthyretin, which together comprise 94% of the total proteins in human serum. Using the manufacturer’s protocol, the low-abundance proteins containing flow-through fractions were collected prior to buffer exchange. Spin concentrators, 5 K MWCO (molecular weight cutoff) with 4 mL capacity from Agilent Technologies (Santa Clara, CA, USA), were used to concentrate the samples, followed by resuspension in 50 mM ammonium bicarbonate buffer. To determine the protein concentration in the depleted samples, a protein [19] assay was performed.

### 2.4. Tryptic Digestion of Low-Abundance Proteins

A 50 µg aliquot of low-abundance proteins was diluted with 50 mM ammonium bicarbonate to make a final volume of 50 µL. The proteins were denatured in a 90 °C water bath for 15 min. The denatured proteins were reduced by adding 200 mM DTT while keeping a DTT to sample volume ratio of 1:40, followed by incubation at 60°C for 45 min. IAA was added to the samples at a 1-to-10 ratio (*v/v*) and incubated at 37 °C for 45 min to perform alkylation. The excess IAA was quenched by adding DTT to the samples at a 1:40 ratio (*v/v*), followed by incubation at 37°C for 30 min. The pH of the reduced and alkylated samples was ensured to be around 8 prior to the tryptic digestion. The enzymatic digestion was performed by adding trypsin/Lys-C to the samples at protein mass ratio of 1:25 (*w/w*) and incubating at 37°C for 18 h. To terminate the tryptic digestion, enough formic acid was added to the samples to make the final concentration of FA 0.5%. The samples were centrifuged at 14,800 rpm for 10 min to precipitate the unwanted large compounds. The glycopeptide-containing supernatant was collected and dried in speed vacuum evaporator.

### 2.5. HILIC Enrichment of N-glycopeptides in NT1 Samples

After tryptic digestion, the glycopeptide samples were enriched using TopTip polyhydroxyethyl A (HILIC) spin columns (Glygen, Columbia, MD, USA). The purpose of this enrichment was to selectively enrich the low-abundance glycopeptides and remove other interfering non-glycopeptides from the samples. A loading buffer containing 80% ACN, 20% H_2_O, 1% TFA, and an elution buffer containing 100% H_2_O and 0.1% TFA were prepared. The dried tryptic digested samples were resuspended in 50 µL of loading buffer. The TopTip HILIC spin columns were washed with 100 µL HPLC-grade H_2_O and conditioned with 100 µL of loading buffer followed by spinning down at 1000× *g* for 1 min. Both washing and conditioning were performed three times. Then, the samples were loaded onto the spin columns and spun down at 1000× *g* for 1 min. The flow-through was loaded onto the column and spun down two more times. The spin columns with samples were then washed three times with 100 µL of loading buffer. Finally, the glycopeptides were eluted from the samples using 50 µL of elution buffer and collected in a separate tube. The collected samples were dried and resuspended in a solution of 98% H_2_O, 2% ACN, and 0.1% FA for LC-MS/MS analysis.

### 2.6. N-Glycopeptide Profiling of NT1 Samples

LC-MS/MS analysis was performed in a Dionex 3000 UltiMate Nano LC system (Thermo Scientific, Sunnyvale, CA, USA) coupled with an Orbitrap Fusion Lumos Tribrid Mass Spectrometer (Thermo Scientific, San Jose, CA, USA). An amount of 2 µg of each glycopeptide sample was injected into the LC system for separation. The samples were passed through a C18 Acclaim PepMap trap column (75 µm × 20 mm, 3 µm, 100 Å, Thermo Scientific, Sunnyvale, CA, USA) for cleaning and online purification prior to separation in a C18 Acclaim PepMap RSLC column (75 µm × 150 mm, 2 µm, 100 Å, Thermo Scientific, Sunnyvale, CA, USA). A 135 min 0.3 µL/min multistage gradient flow was used to perform the separation of the glycopeptides. The mobile phase A was 98% H_2_O, 2% ACN, 0.1% FA; the mobile phase B was 100% ACN and 0.1% FA. The column oven temperature was set to 60 °C. In the first 10 min of the run, the mobile phase B was kept constant at 5% and then ramped to 30% over the next 85 min. The mobile phase B was increased from 30% to 50% in 95–110 min, 50% to 90% in 110–113 min, and then kept constant at 90% for 5 min. Then, the mobile phase was decreased to the initial stage, i.e., 5%, and kept constant until the end of the run.

The separated *N*-glycopeptides were sent to the Orbitrap Fusion Lumos Tribrid Mass Spectrometer via a nano electrospray ionization source at 1.6 kV in positive ion mode. The analysis was performed in a data-dependent acquisition (DDA) mode. The full MS scan was performed with a scan range of 350–1800 *m*/*z*, and the analytes were detected in the Orbitrap detector at a resolution of 120,000. The 10 most intense ions were selected for MS2 fragmentation in a high-energy collision dissociation (HCD) cell, with 25% of normalized collision energy (NCE) at a resolution of 60,000. The acquired data were processed using Glyco-Decipher v1.0.3 software. The Homo sapiens database file was used as reference. The identification of glycopeptides was conducted at a mass tolerance of 10 ppm, maximum missed cleavages of two, peptide length 6–40, peptide mass range 600–4500 Da, and charged state 2–6. The fixed modification was set for the carbamidomethylation of cysteine and the variable modification for the oxidation of methionine and acetylation in the N-termini of the peptides.

### 2.7. Parallel Reaction Monitoring (PRM) Validation of the Obtained Glycoppeides in NT1 Samaples

To validate the results of the glycoproteomic analysis, a targeted PRM analysis was performed, particularly on the statistically significant glycopeptides. A sample was analyzed to make a transition list of glycopeptide precursor ions, with their corresponding retention time as well as fragment ions of the precursors. The sample was analyzed on a Dionex 3000 UltiMate Nano LC system (Thermo Scientific, Sunnyvale, CA, USA) connected to a Q Exactive HF mass spectrometer (Thermo Scientific). The LC gradient was the same as the gradient used for untargeted analysis. The data were acquired in positive ion mode. The following parameters were set for PRM analysis: resolution 30,000, a HCD normalized collision energy (NCE) of 25 %, AGC (Automatic Gain Control) target 2e5, maximum injection time 100 milliseconds, and isolation window 4.0 *m*/*z*. A larger isolation window could help to ensure that all target ions are selected and fragmented, as described by Defossez et al. [42]. The data were processed manually using Thermo Scientific Xcalibur software. To find the statistically significant glycopeptides (*p* value < 0.05) based on their relative abundance, a nonparametric Mann–Whitney U-test was performed using GraphPad Prism 9.3.1 (GraphPad Software Company, La Jolla, CA, USA). 

## 3. Results

The proteomic analysis of narcolepsy type 1 samples was published in our previous work [43]. This analysis identified several significant proteins, such as inter-alpha-trypsin inhibitor H4, complement factor B, ceruloplasmin, fibronectin, and prothrombin. These proteins were found to play important roles in biochemical pathways that are correlated with narcolepsy type 1 [43]. Hence, it was logical to conduct a focused investigation on NT1 glycoproteomics and examine the expression of noteworthy glycopeptides inside two distinct cohorts: the control group and the NT1 group. 

### 3.1. Analytical Approach of NT1 Serum Samples 

**Figure 1:**
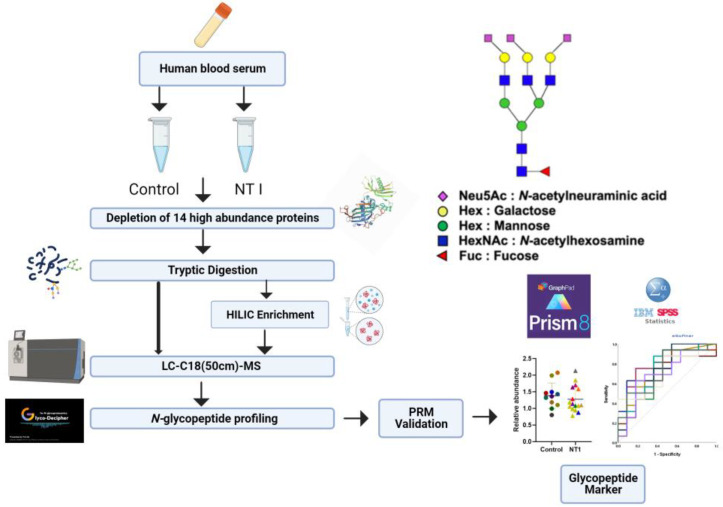
Workflow for LC-MS/MS analysis. Symbols: blue squares, *N*-acetylglucosamine (GlcNAc); yellow circles, galactose (Gal); red triangles, fucose (Fuc); green circles, mannose (Man); blue circles, glucose (Glc); purple diamonds, *N*-acetylneuraminic acid (NeuAc/sialic acid).

Initially, a total of 27 serum samples, including 16 NT1 samples and 11 healthy controls, were subjected to depletion using a Human 14 Multiple Affinity Removal Column. This column depletes the 14 high-abundance human serum proteins/glycoproteins, namely albumin, IgG, antitrypsin, IgA, transferrin, haptoglobin, fibrinogen, alpha2-macroglobulin, alpha1-acid glycoprotein, IgM, apolipoprotein AI, apolipoprotein AII, complement C3, and transthyretin (Agilent Technologies Inc.). Removal of these proteins effectively expands the dynamic range of the low-abundance serum proteins in LC-MS analysis [44]. As a result, an LC-MS/MS approach was applied for the quantitation of intact *N*-glycopeptides released from low-abundance serum samples to study the variation of the *N*-glycoproteome between control and NT1 patients. The experimental workflow is shown in Figure 1. Briefly, the depleted samples were subjected to denaturation at 90 °C for 15 min. Then, the denatured proteins were reduced, alkylated, and digested using trypsin. Finally, samples were divided into two parts: one underwent C18 (50 cm)-LC-MS/MS analysis without enrichment and was analyzed by LC-MS/MS, whereas the other was subjected to HILIC enrichment prior to LC-MS glycoproteomic analysis (Figure 1). The C18 (50 cm)-LC-MS /MS was applied in DDA mode to identify the *N*-glycopeptides present in the samples. 

### 3.2. Investigation of N-glycopeptide Abundance and Distribution in NT1 Samples 

**Figure 2:**
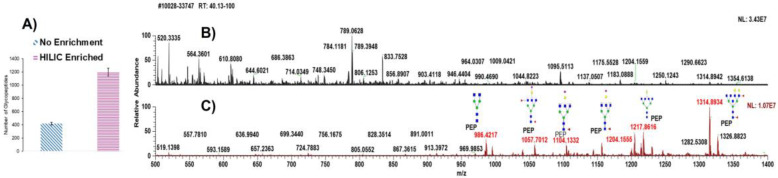
HILIC enrichment and no enrichment comparison of *N*-glycopeptides: (**A**) Number of identified glycopeptides without and with enrichment, blue and pink color, respectively, (the error bar represents the SD calculated from the average number of glycopeptides in a biological replicate). (**B**) MS spectra of non-enriched *N*-glycopeptides from NT1 samples. (**C**) MS spectra of enriched *N*-glycopeptides from NT1 samples. Symbols are described in Figure 1.

First, the samples’ raw files were individually analyzed using Glyco-Decipher software to make an in-common preliminary list. Then, structures were manually confirmed based on monoisotopic mass, charge, retention time, and MS2 spectra. Two groups from the enriched and non-enriched analysis showed important differences in the identification of glycopeptides. Before enrichment, 417 *N*-glycopeptides were identified; after HILIC enrichment, 1188 glycopeptides were identified (Figure 2A). The biological replicates used in this investigation were used to demonstrate the differences between the two approaches. The standard deviation of non-enriched samples was 56, whereas the standard deviation of enriched glycopeptides was 92, which is presented as an error bar in this figure. The identified glycopeptides varied across the analyzed samples. It is also important to consider that since the purpose of the experiment is to identify as many glycopeptides as possible, enrichment is clearly beneficial. In addition to enhancing the number of glycopeptides, MS spectra were also used to compare two methods in the same sample. As shown in Figure 2B, without the enrichment of samples, few signals corresponding to the glycopeptides were detected and no signals of significant glycopeptides were detected. However, in the HILIC-enriched samples (Figure 2C) significant glycopeptides were assigned and the glycopeptides’ intensities were enhanced considerably. This increase was likely activated by the depletion of non-glycosylated peptides [45]. The majority of glycopeptide signals have higher molecular masses than the peptides in the non-depleted samples, which corresponds to the mass contribution of the attached glycans.

**Figure 3:**
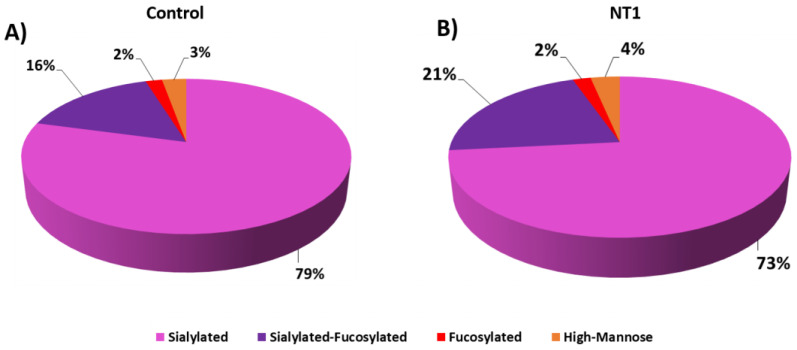
Changes in *N*-glycan distribution including sialylated, sialylated–fucosylated, fucosylated, and high mannose in percentages based on abundance of glycopeptides for (**A**) control and (**B**) narcolepsy type 1 samples.

The abundance of glycopeptide types was also studied, and the glycome changes associated with the groups of glycan types are presented in Figure 3. The four main glycan groups, sialylated, sialylated–fucosylated, fucosylated, and high mannose, were investigated in two cohorts. The most abundant group was sialylated, with around 80% of the total glycan types. From the control in Figure 3A to NT1 in Figure 3B, the abundance of sialylated glycopeptides decreased by 6%, sialylated–fucosylated structures increased by 5%, and the abundance of high mannose structures increased by 1%. There was no significant difference in the amount of fucosylated groups between the two groups of samples. The comparison between sialylated and sialylated–fucosylated groups demonstrates a statistically significant difference, as indicated by a *p* value of 0.02. The different abundances between the two cohorts are not statistically significant for the fucosylated and high mannose groups. Upon in-depth examination, it was observed that the majority of the noteworthy glycopeptides were found in the sialylated groups.

### 3.3. Comparison of Control and NT1 Analysis of Identified Glycopeptides

**Figure 4:**
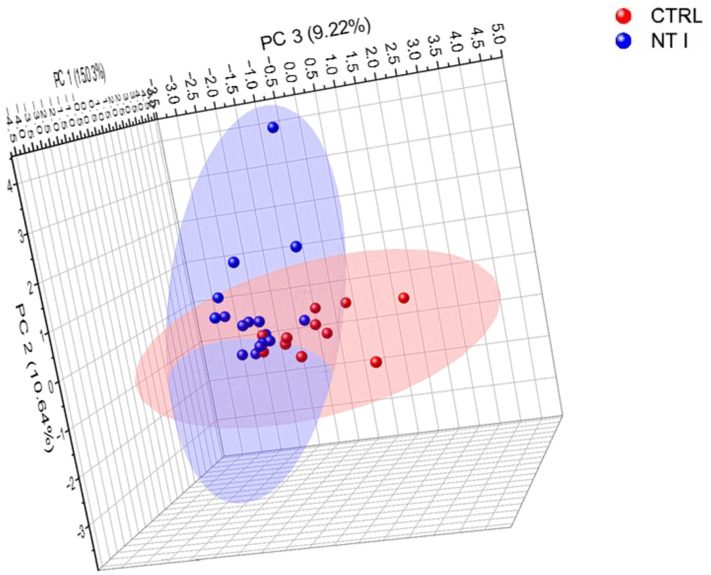
Unsupervised principal component analysis (PCA) at 95% confidence level for total identified glycopeptides.

Unsupervised PCA was used to show differential expression between NT1 and healthy cohorts for enriched glycopeptides, as shown in Figure 4. The PCA was generated with Origin2022b software at confidence level of 95% for all 1188 enriched glycopeptides, which showed an acceptable level of clustering. The results indicated differential expression between non-disease- and disease-state groups. The variation in expression levels of the glycopeptides in NT1 samples with control samples was expected, which was clearly demonstrated in PCA. In the first dimension, PC1 is 15.03%, PC2 is 10.64%, and PC3 is 9.22%. According to the percentages given, PC1, PC2, and PC3 account for a sizable chunk of the difference in the levels of glycopeptide expression in the NT1 samples and control samples. The most variation is explained by PC1, followed by PC2 and PC3. That these three dimensions were employed to depict the data’s variability shows they are adequate to account for the data’s most significant patterns. The data’s dimensionality may be reduced, making it simpler to display and interpret. The points in Figure S1 reflect the individual samples at each location. To indicate which sample differs the most from the others and which samples overlap at other locations, we labeled each point with the number of samples from 1 to 27 in two colors. Each sample’s number was used to plot the PCA in Figure S1, and the legend identifies each sample. NT1 is represented by the blue points and the control samples by the red points. There is minimal overlap between sample 1 from the control group and sample 23 from NT1, suggesting that there is no substantial clustering in abundance when considering the total identified glycopeptides in these two samples. Additionally, there is no discernible difference between sample 9 from the control group and 26 from the NT1 group. In contrast, samples 10, 11, 7, 3, and 4, taken from healthy individuals, are far from the disease samples. When comparing the two cohorts, we were unable to obtain a sufficient amount of clustering between the male and female gender groups. However, unsupervised PCA concluded that the two sample groups used in this study are sufficiently clustered.

### 3.4. Differently Expressed Relative Abundance of N-glycopeptides Derived from Low-Abundance Serum Glycoproteins in Control and NT1 Samples 

**Figure 5:**
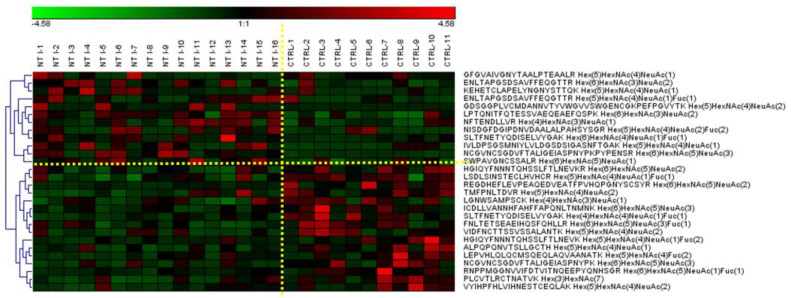
Heatmap of *N*-glycopeptides with statistically significant expression structures between control and NT1 samples.

The *N*-glycopeptide peak area obtained from the Glyco-Decipher software [46] was used to calculate the relative abundances of the identified structures shown in Table S1. The data were further analyzed by GraphPad Prism 9.3.1 (GraphPad Software Company, La Jolla, CA, USA). A Mann–Whitney U-test was employed to find statistically significant *N*-glycopeptides (*p* values < 0.05) that differentiated between the control and NT1 samples. Twenty-eight *N*-glycopeptides from low-abundance glycoproteins showed a statistically significant difference in expression between the control and disease groups. A heatmap was used to show up-regulation and down-regulation of the *N*-glycopeptide structures (Figure 5). A total of 17 of the significant *N*-glycopeptides were observed as down-regulated in NT1, whereas 11 structures were up-regulated. The red color represents up-regulated and the green color down-regulated *N*-glycopeptides. 

The statistical results with a cut-off AUC (area under the ROC (receiver operating characteristic) curve) of 0.75 are described in Table 2. A difference of 28 significant glycopeptides between the NT1 and control samples is important to note, since some of them have large differences in abundance and identification and could be potential biomarkers in NT1 samples. To simplify the annotation of glycan structures, a four-digit nomenclature was employed (as in Table 2), where each digit represents the number of monosaccharides associated with an *N*-glycan structure in the following order: *N*-acetylhexosamine, hexose, fucose, and *N*-acetylneuraminic acid (HexNAc, Hex, Fuc, and Neu5Ac). The most remarkable structures are sialylated, which follows the note mentioned in Section 3.2. Among the up-regulated glycopeptides, ENLTAPGSDSAVFFEQGTTR + 5-4-1-1 and 6-3-0-2 derived from CP glycoprotein and glycopeptide LPTQNITFQTESSVAEQEAEFQSPK + 6-3-0-2 derived from ITIH4 glycoprotein, which increased in the NT1 samples, were observed as prominent alternations in expression the were associated with a change in the narcolepsy patient.

**Figure 6:**
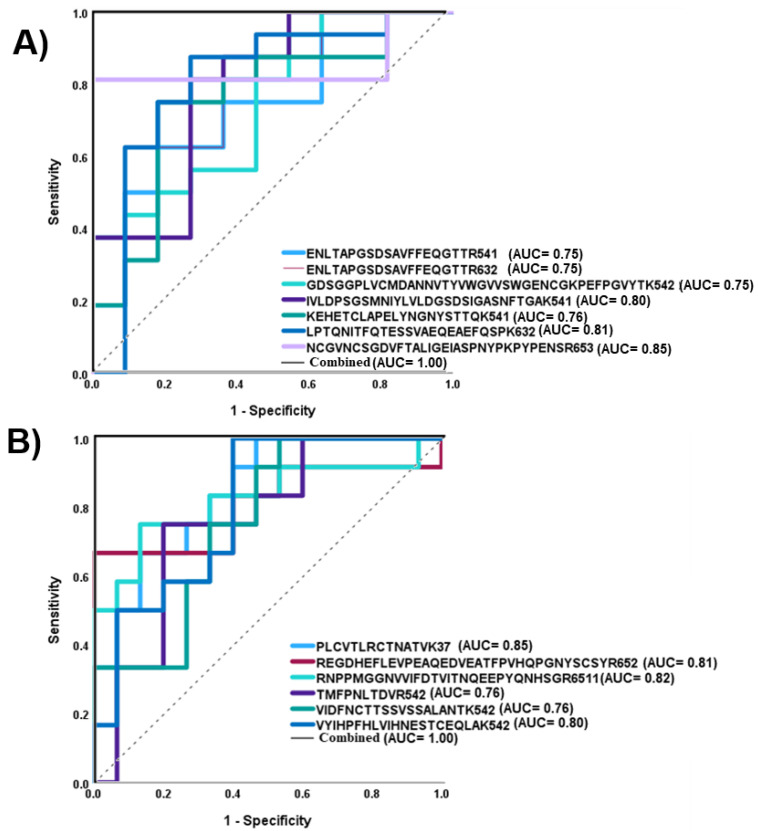
ROC curves of the *N*-glycopeptides with statistically significant differences between control and NT1 samples: (**A**) up-regulated glycopeptides; (**B**) down-regulated glycopeptides.

The performances of statistically significant glycopeptides were also assessed by applying ROC curves (Figure 6). The results are presented in two parts: Figure 6A shows the up-regulated and Figure 6B shows the down-regulated *N*-glycopeptides with a cut-off AUC value of 0.75. We observed seven up-regulated *N*-glycopeptides, including mono, di, and three sialylated *N*-glycopeptides, and six down-regulated *N*-glycopeptides, including sialylated, high mannose, and fucosylated glycopeptides. The up-regulated structure NCGVNCSGDVFTALIGEIASPNYPK + 6-5-0-3 with an AUC value of 0.85 and a *p* value of 0.02 and the down-regulated glycopeptide PLCVTLRCTNATVK + 3-7-0-0 with an AUC value of 0.85 and a *p* value of 0.01 were the best structures for showing differentiation between disease and healthy groups based on AUC. In addition, the signals of the up- and down-regulated *N*-glycopeptides were combined to gain sensitivity and specificity. This evaluation can be performed by applying binary logistic regression from SPSS software. For both up- and down-regulated *N*-glycopeptides, the AUC was enhanced to 1.00, which is shown in Figure 6 as “combined” on the plots .

To further examine the distinction between the two cohorts, dot plots were employed (Figure S2). The plots show changes in the two groups for the NT1 and control samples. In di-sialylated glycopeptides, with VIDFNCTTSSVSSALANTK + 5-4-0-2 and a *p* value of 0.01, the glycopeptide’s abundance was lower in control vs. NT1 samples. The glycopeptide SLTFNETYQDISELVYGAK + 4-4-1-1 also showed a decrease in the abundance of the structure from control to disease, with a *p* value of 0.007. For the next complex structure, LPTQNITFQTESSVAEQEAEFQSPK + 6-3-0-2, with *p* value of 0.008, the quantity in healthy control samples was higher than in the disease samples. These results correlated with the heatmap reported in Figure 5.

### 3.5. N-glycopeptide Isomer Quantification 

Since site-specific analysis of isomeric glycopeptides is challenging, the utilization of a 135-minute gradient for the C18 (50cm) column has demonstrated efficacy in the investigation of isomeric separation [47,48]. We analyzed isomers in two groups of samples and quantified them manually. The peak area was calculated for the single isomeric peak in the two groups of the control and NT1 samples individually. Ten *N*-glycopeptides were found to have the most statistically significant differences between two cohorts (Table 3). The glycopeptide ALPQPQNVTSLLGCTH + 6-4-1-1 isomers 1 and 2, with *p* values of 0.006 and 0.001, respectively, and the glycopeptide FNLTETSEAEIHQSFQHLLR + 7-6-0-4 isomer 2, with a *p* value of 0.0005 and an AUC of 0.84 showed the most notable differences in abundance between the two groups of the control and NT1 samples. The ROC/AUC values and the precursor mass of isomers are reported in Table 3. Among the significant isomers, two of them that exhibited significant variations in isomer abundance between the two distinct cohorts are displayed in Figure S3. The glycopeptide LDAPTNLQFVNETDSTVLVR + 6-5-0-3 isomer 2 and FNLTETSEAEIHQSFQHLLR + 7-6-0-4 isomers 1 and 2 had *p* values of 0.001, 0.02, and 0.0005, respectively. In addition, isomers for four other structures are presented in Figure S4; even though some of them were not resolved completely, they could still be analyzed later by developing methods and LC-MS/MS parameters.

### 3.6. Validation Strategy of Targeted Glycoproteomics (LC-PRM-MS/MS)

A targeted PRM approach was applied to validate some *N*-glycopeptides based on the highest levels of fold change. Thirty glycopeptides were verified, and seven of the *N*-glycopeptides showed the same trend of fold change. Four of the validated structures were up-regulated and the three were down-regulated in the NT1 samples. Three significant *N*-glycopeptides were among the validated glycopeptides. ALPQPQNVTSLLGCTH + 5-4-0-1, KEHETCLAPELYNGNYSTTQK + 5-4-0-1, and VYIHPFHLVIHNESTCEQLAK + 5-4-0-2 had fold changes of 0.95, 1.02, and 0.97, respectively. In Table 4, the list of validated *N*-glycopeptides with untargeted and targeted (PRM) fold change values is reported.

## 4. Discussion

In this study, we employed the technique of enriched *N*-glycopeptide profiling for identification of potential serum biomarkers extracted from the blood sera of healthy donors and NT1 patients. To understand NT1, a disease that has not yet been fully investigated, it is crucial to discover the glycosylation sites in glycoproteins in disease samples and therefore increase prediction for its diagnosis [4]. Due to a meaningful relationship between human leukocyte antigen (HLA) and NT1 [49], patient samples with existing HLA typing were chosen for this study. The focus of the present study was to profile the *N*-glycopeptides derived and enriched from low-abundance serum glycoproteins, because the wide dynamic range of high-abundance serum proteins would cover the presence of the low-abundance proteins that play a prominent role in the development of numerous diseases [50,51,52] and, more specifically, for NT1 patients in this study. High-abundance glycoproteins, including IgG, antitrypsin, IgA, transferrin, haptoglobin, fibrinogen, alpha-2-macroglobulin, alpha1-acid glycoprotein, IgM, apolipoprotein AI, apolipoprotein AII, complement C3, and transthyretin, were removed to obtain the low-abundance glycoproteins. To complete the depletion of high-abundance proteins, depletion with an Agilent column was used. For low-abundance glycoproteins, the study focused on HILIC-enriched glycopeptides whose number and quantity had been enhanced. As presented in Figure 1, the samples were prepared in two parts: one without enrichment and the other with HILIC enrichment. The intensity and the number of identified glycopeptides were compared. Both groups of samples were analyzed using Glyco-Decipher, and during the comparison of enriched and non-enriched glycoproteins, we found more than twice the number of *N*-glycopeptides in the HILIC-enriched NT1 samples that were presented in Figure 2. Depletion of non-glycosylated glycopeptides has been shown to increase the number of glycopeptides [45]. Additionally, it was reported that using TFA as an ion pairing reagent in the recovery solution during enrichment can help to neutralize the charge of peptides, increase hydrophobicity, and improve the enrichment of glycopeptides [45,53]. Supplementary Figure S5A shows the fragmentation of the AALAAFNAQNNGSNFQLEEISR glycosylation site in the NT1 samples obtained via HCD MS/MS. B, Y, and b/y ions are observed in different colors in the spectrum and facilitated confident structural assignment. 

Glycan heterogeneity was also shown in Figure S5B, in which di-sialylated glycan 5-4-0-2 had the highest number of glycopeptide spectrum matches. Since the HILIC enrichment method showed progression in the glycopeptide identification and abundance of significant structures, a study of the reproducibility of the method is also important. Therefore, technical replicates of the samples and the MS spectra of four glycopeptides are shown in Figure S6. The similarity of the spectra coming from the duplicates proved that the HILIC enrichment method was highly reproducible. In the different types of glycans in glycopeptides, the most representative *N*-glycopeptide types were, in order from highest to lowest: sialylated, sialylated–fucosylated, fucosylated, and high mannose (Figure 3). In the changes of glycan type, we observed a decreasing abundance of sialylated *N*-glycopeptide types and, conversely, an increasing abundance of sialylated–fucosylated *N*-glycopeptides from control to NT1 samples. 

When there is a high abundance of sialylated glycopeptides in the NT1 samples, introducing bias to the HILIC enrichment method is inevitable. One potential bias that may arise from HILIC enrichment is the preferential enrichment of glycopeptides containing specific types of glycans. For instance, previous studies have demonstrated that HILIC materials exhibit a greater binding preference toward glycopeptides containing sialylated glycans [54]. This may cause some types of glycopeptides with particular types of glycans to be underrepresented in the enriched sample. Thus, we processed the number of *N*-glycopeptides in different types of glycans, both enriched and non-enriched, and the results showed sialylated glycopeptides to be the most abundant glycopeptides in both samples of glycopeptides. The results of the comparison of these two methods from different types of glycans attached to peptides appear in Figure S7. Sialylated glycopeptides demonstrated the highest abundance of structures even without HILIC enrichment. Other types of structures were also increased by employing enrichment. Therefore, no specific type of glycopeptide was advanced by the HILIC method. Another potential bias introduced by HILIC enrichment is that it can favor the enrichment of glycopeptides from certain proteins [54]. For example, HILIC materials have been shown to have a higher affinity for glycopeptides from mucins than for glycopeptides from other types of proteins. This can lead to an overrepresentation of glycopeptides from mucins in an enriched sample. As explained in Table 2, different proteins were present in the HILIC-enriched NT1 samples as sources of glycopeptides. 

The unsupervised PCA in Figure 4 was used to show the reasonable separation of two groups of samples. The 3D PCA was performed using Origin with 95% confidence. In addition, we applied a Mann–Whitney U-test to find *N*-glycopeptides that were significantly unique and expressed notable differences between the control and NT1 samples. The *p* value < 0.05 obtained from GraphPad was employed to differentiate the sample groups. 

Supplementary Table S1 shows the quantification values of samples used for the calculation of the *p* values. Twenty-eight unique *N*-glycopeptides were significantly expressed when we compared the two groups of samples. Eleven of the structures were observed to be up regulated and seventeen of the glycopeptides were decreased in NT1 samples. The *p* values of significant glycopeptides are shown in Table 2. The protein sources of significant glycopeptides were already reportedly involved in immunological activities, cancer, and neurodegenerative disease [43,55]. Inter-alpha-trypsin inhibitor H4, complement factor B, ceruloplasmin, fibronectin, and prothrombin were among the significant glycoproteins that were identified in the 28 significant glycopeptides. The red color and the green color on the heat map (Figure 5) represent up-regulated and down-regulated glycopeptides, respectively. An additional assessment was obtained through an ROC curve (Figure 6). The *N*-glycopeptides that were revealed to be up-regulated (7) and down-regulated (6) had high AUC values of more than 0.75. Although the AUC values ranged from 0.75 to 0.85, after a combination of two analyses in binary logistic regression from SPSS software, the AUC value increased to 1.0 (Figure 6). Dot plots (Figure 3S) showed distinct differentiation in the two groups of samples. For example, VIDFNCTTSSVSSALANTK + 5-4-0-2 and SLTFNETYQDISELVYGAK + 4-4-1-1, with *p* values of 0.01 and 0.007, respectively, showed meaningful differences between the healthy and disease samples. C18-LC-MS/MS with a long gradient of over 135 min revealed isomers in sialylated glycopeptides. Although some of the isomers were not completely resolved, we could identify 11 isomers that showed significantly different expression between the two cohorts (Table 3). The use of the PGC column could possibly identify the isomers of glycopeptides more clearly [30]. The role of glycan isomers was studied in different types of disease and cancer [12,56,57]; however, no research has explained how important the glycan isomers in the NT1 samples are. Because the differential expression of isomers of *N*-glycopeptides between the two groups of samples is significant as a potential narcolepsy biomarker, we endeavored to evaluate a few isomers that showed different abundances between the control and NT1 groups (Figure S3). Due to the fact that the most common structures contained sialic acid, the most common isomers were predominantly mono- and di-sialylated *N*-glycopeptides. From 108.6 min to 110 min, the sialylated glycopeptide with the YPHKPEINSTTHPGADLQENFCR backbone exhibited three isomers. The sialylated–fucosylated glycopeptide with the NISDGFDGIPDNVDAALALPAHSYSGR backbone exhibited two isomers (Figure S4). 

As a validation, LC-PRM-MS/MS analysis was performed for the most important identified *N*-glycopeptides. The selection accorded to the level of fold change. Among 30 verified *N*-glycopeptides, we found 7 structures that showed the same trend of fold change: 4 structures were up-regulated and 3 *N*-glycopeptides were down-regulated in the NT1 samples. Overall, the combined impact of hereditary and epigenetic variables, which undoubtedly interact within the pathophysiological pathways of NT1, is crucial to emphasize in our glycoproteomic analysis [58]. As previously stated, the predominant *N*-glycopeptide seen in NT1 samples is the sialylated type. Notably, this particular form of glycosylation has been linked to the immunological response and inflammation [59,60,61]. The role of sialylated glycans was discussed by the Bhide group with regard to cancer. They observed that cancer cell lines exhibited an altered glycosylation pattern, which could aid cancer cell survival, drug resistance, and metastasis [61,62,63]. However, there is currently no research available on the presence of particular glycans and glycopeptides for NT1. Understanding the role of sialylated glycans in inflammation could lead to new therapeutic strategies for a variety of inflammatory diseases. As described in Table 2, the glycopeptides LPTQNITFQTESSVAEQEAEFQSPK + 6-3-0-2, with a 0.008 *p* value and a 0.81 AUC derived from the ITH4 gene; IVLDPSGSMNIYLVLDGSDSIGASNFTGAK + 5-4-1-0, with a 0.01 *p* value and a 0.80 AUC derived from CFB; and ENLTAPGSDSAVFFEQGTTR + 5-4-1-1 from the CP gene, with a 0.03 *p* value and a 0.75 AUC are among the significant glycopeptides that would be potential biomarkers for NT1. The proteins listed in the study have previously been reported as major genes that exhibit alterations in expression in NT1 samples [43]. In this investigation, these glycoproteins were further verified.

By analyzing the glycosylation patterns of serum proteins in narcoleptic individuals, researchers can potentially uncover biomarkers that are indicative of the autoimmune processes underlying the disease. As reported in this study, aberrant glycosylation profiles may pinpoint specific glycoproteins or immune-related molecules implicated in NT1’s pathogenesis, contributing to the understanding of the autoimmune mechanisms responsible for orexin neuron destruction and providing a more comprehensive understanding of the disorder’s biological basis.

The clinical implications of assessing serum *N*-glycopeptides in narcolepsy research are significant. The identification of glycosylation-related biomarkers in serum could aid in the early diagnosis and risk assessment of NT1, allowing for timely intervention and improved patient outcomes. Additionally, a better grasp of the glycoproteomic alterations associated with narcolepsy might guide the development of novel therapeutic strategies, such as immune-modulating treatments aimed at preventing or mitigating orexin neuron loss. This approach holds the potential to improve narcolepsy management, offering tailored therapies that address the underlying autoimmune component and provide more effective symptom control. In summary, the assessment of serum *N*-glycopeptides offers a promising avenue for advancing both our understanding of NT1 biological intricacies and the development of targeted clinical interventions for this challenging neurological disorder.

## Figures and Tables

**Table 1:** Clinical information of NT1 patients and healthy controls.

Information	Control	NT1
Sample size	11	16
Gender (M/F)	7/4	11/5
Age ranges (year)	28–73	19–71
Orexin level in CSF (pg/mL)	Not available	0–119.6
HLA1 DQB1^1^0602 allele	2	15
Body mass index	Not available	20.2–28.6

^1^ Human leukocyte antigen.

**Table 2:** Glycopeptides with statistically significant changes in abundance between control and NT1 samples and the AUC values obtained from ROC analysis. Glycan nomenclature: HexNAc, Hex, Fuc, and NeuAc (*N*-acetylhexosamine, hexose, fucose, and *N*-acetylneuraminic acid).

Glycopeptides	Protein Name	*p* Value	ROC/AUC
ENLTAPGSDSAVFFEQGTTR + 5-4-1-1	Ceruloplasmin	0.03	0.75
ENLTAPGSDSAVFFEQGTTR + 6-3-0-2	Ceruloplasmin	0.03	0.75
GDSGGPLVCMDANNVTYVWGVVSWGENCGKPEFPGVYTK + 5-4-0-2	Inter-alpha-trypsin inhibitor heavy chain H4	0.03	0.75
IVLDPSGSMNIYLVLDGSDSIGASNFTGAK + 5-4-1-0	Complement factor B	0.01	0.80
KEHETCLAPELYNGNYSTTQK + 5-4-0-1	Coagulation factor XIII B chain	0.02	0.76
LPTQNITFQTESSVAEQEAEFQSPK + 6-3-0-2	Inter-alpha-trypsin inhibitor heavy chain H4	0.008	0.81
NCGVNCSGDVFTALIGEIASPNYPK + 6-5-0-3	Complement C1s	0.02	0.85
REGDHEFLEVPEAQEDVEATFPVHQPGNYSCSYR + 6-5-0-2	Alpha-1B-glycoprotein	0.006	0.81
RNPPMGGNVVIFDTVITNQEEPYQNHSGR + 6-5-1-1	Alpha-1B-glycoprotein	0.01	0.82
VIDFNCTTSSVSSALANTK + 5-4-2-0	Histidine-rich glycoprotein	0.01	0.76
PLCVTLRCTNATVK + 3-7-0-0	Angiotensinogen	0.01	0.85

**Table 3:** Glycopeptides isomers with statistically significant changes in abundance between control and NT1 samples.

Glycopeptides	*p* Value	Precursor Mass	ROC/AUC
LHINHNNLTESVGPLPK + 6-5-1-1 Isomer 1	0.009	985.919	0.88
ELHHLQEQNVSNAFLDKGEFYIGSK + 6-5-2-2 Isomer 1	0.01	1213.826	0.66
FNLTETSEAEIHQSFQHLLR + 7-6-0-4 Isomer 1	0.02	1203.824	0.72
FNLTETSEAEIHQSFQHLLR + 7-6-0-4 Isomer 2	0.0005	1203.824	0.84
ALPQPQNVTSLLGCTH + 6-4-1-1 Isomer 1	0.006	1006.766	0.84
ALPQPQNVTSLLGCTH + 6-5-0-2 Isomer 1	0.02	1152.453	0.81
NHSCSEGQISIFR + 5-4-0-2 Isomer 1	0.02	925.7962	0.65
GLNVTLSSTGR + 5-4-2-1 Isomer 1	0.003	1057.447	0.69
LDAPTNLQFVNETDSTVLVR + 6-5-0-3 Isomer 2	0.001	1326.211	0.70
ALPQPQNVTSLLGCTH + 6-4-1-1 Isomer 2	0.001	1006.766	0.71

**Table 4:** List of glycopeptides validated by LC-PRM-MS/MS including fold change before and after validation by PRM.

Glycopeptides	Protein Name	FC	FC (PRM)
ALPQPQNVTSLLGCTH + 5-4-0-1	HPX	0.42	0.95
KEHETCLAPELYNGNYSTTQK + 5-4-0-1	Coagulation factor XIII B chain	2.88	1.02
VYIHPFHLVIHNESTCEQLAK + 5-4-0-2	Angiotensinogen	0.42	0.97
SPYYNVSDEISFHCYDGYTLR + 4-3-0-1	Complement factor B	1.17	1.03
YPHKPEINSTTHPGADLQENFCR + 5-4-1-1	F2(Prothrombin)	1.47	1.15
YPHKPEINSTTHPGADLQENFCR + 6-6-00	F2	0.86	0.75
YPHKPEINSTTHPGADLQENFCR + 7-5-2-0	F2	1.13	1.72

## Data Availability

The mass spectrometry proteomics data have been deposited in the ProteomeXchange Consortium via the identifier PXD043648 (doi: 10.25345/C5FF3M94K). The data were submitted via the MassIVE partner repository. Reviewer username: MSV000092391_reviewer, URL: ftp://MVS000092391@massive.ucsd.edu.

## Supplementary Materials

The following supporting information can be downloaded at: https://www.mdpi.com/article/10.3390/biom13111589/s1, Table S1: The normalized values of significant *N*-glycopeptides in two groups of control and NT1 samples. Figure S1: Unsupervised PCA for total identified glycopeptides in control and NT1 samples including each sample number. Figure S2: Dot plots of the *N*-glycopeptides with statistically significant differences between the control and NT1 samples. Figure S3: Two significant isomers derived from control and NT1 blood serum samples Figure S4: Isomeric *N*-glycopeptides observed with the C18 (50cm) column Figure S5: Fragments of *N*-glycopeptides in NT1 including Y, B, and b/y ions in NT1 samples and Glycan heterogeneity on the glycosylation site. Figure S6: MS spectra from three technical replicates in NT1 samples for 4 *N*-glycopeptides to display the reproducibility of the enrichment method. Figure S7: Comparing a number of different types of glycan in the NT1 samples by the two method, without enrichment and with HILIC enrichment.

## Abbreviations

ABC, ammonium bicarbonate; ACN, acetonitrile; NT1, narcolepsy type 1; DTT, dithiothreitol; HLA, human leukocyte antigen; FA, formic acid; IAA, iodoacetamide; IPA, ingenuity pathway analysis; PCA, principal component analysis; HPLC, high-performance liquid chromatography; HILIC, hydrophilic interaction liquid chromatography; SD, standard deviation; TFA, trifluoroacetic acid; AUC, area under curve; ROC, receiver operating characteristic.
