# Supplementary material for: LC-MS/MS Quantitation of HILIC-Enriched N-glycopeptides Derived from Low-Abundance Serum Glycoproteins in Patients with Narcolepsy Type 1
Source: Biomolecules. 2023 Oct 28;13(11):1589. doi: 10.3390/biom13111589 (PMC10669497; doi:10.3390/biom13111589)
Supplement: Supplementary file 1 [file biomolecules-13-01589-s001.zip › biomolecules-2625212-supplementary.pdf]

[Supplementary data]

LC-MS/MS Quantitation of HILIC-Enriched *N*-glycopeptides derived from low abundance serum glycoproteins in patients with Narcolepsy Type 1

Mojgan Atashi[1], Cristian D. Gutierrez Reyes[1], Vishal Sandilya[1], Waziha Purba[1], Parisa Ahmadi[1], Md. Abdul Hakim[1], Firas Kobeissy[2,3], Giuseppe Plazzi[4,5], Monica Moresco[4], Bartolo Lanuzza[6], Raffaele Ferri[6], Yehia Mechref[1]*

[1] Department of Chemistry and Biochemistry, Texas Tech University, Lubbock, TX, USA; mo-jgan.atashi@ttu.edu (MA); Cristian.d.gutierrez-reyes@ttu.edu (CDGR); Vishal.Sandilya@ttu.edu (VS); wpurba@ttu.edu (WP); pahmadi@ttu.edu (PA); md-abdul.hakim@ttu.edu (MAH)
[2] Faculty of Biochemistry and Molecular Genetics, American University of Beirut, Beirut, Lebanon; fkobaissy@msm.edu (FK)
[3] Morehouse Scholl of Medicine (MSM), Center for Neurotrauma, Multiomics &Biomarkers, Depart-ment of Neurobiology, Atlanta, Georgia, USA; fkobaissy@msm.edu (FK).
[4] IRCCS, Instituto delle Scienze Neurologiche di Bologna, Bologna, Italy; giuseppe.plazzi@unibo.it, monica.moresco@ausl.bologna.it (MM)
[5] Department of Biomedical, Metabolic and Neural Sciences, University of Modena and Reggio Emilia, 41125 Modena, Italy; giuseppe.plazzi@unibo.it (GP)
[6] Sleep Research Center, Department of Neurology IC, Oasi Research Institute-IRCCS, Tronia, Italy, blanuzza@oasi.en.it (BL), rferri@oasi.en.it(RF)

* Correspondence: Yehia.mechref@ttu.edu; Tel.: +1 806-742-3059

Table of Contents:

Supplementary Figure S1: Unsupervised PCA for total identified glycopeptides in control and NT1 samples including each sample number

Supplementary Figure S2: Dot plots of the *N*-glycopeptides with statistically significant differences between the control and NT1 samples.

Supplementary Figure S3: Two significant isomers drived from control and NT1 blood serum samples for **A)** LDAPTNLQFVNETDSTVLVR +6-5-0-3, and **B)** FNLTETSEAEIHQSFQHLLR+7-6-0-4

Supplementary Figure S4: Isomeric *N*-glycopeptides observed with the C18 (50cm) column **A)** sialylated glycopeptide with YPHKPEINSTTHPGADLQENFCR backbone, **B)** di-sialylated glycopeptide with LGACNDTLQQLMEVFK backbone, **C)** complex glycopeptide with NISDGFDGIPDNVDAALALPAHSYSGR, and **D)** di- sialylated glycopeptides with LQNNENNISCVER peptide backbone

Supplementary Figure S5: **A**)Fragments of *N*-glycopeptides in NT1 including Y, B, and b/y ions in NT1 sample, **B**) glycan heterogeneity on the glycosylation site

Supplementary Figure S6: MS spectra's from three technical replicates in NT1 samples for 4 N-glycopeptides to display the reproducibility of the enrichment method

Supplementary Figure S7: Comparing a number of different types of glycan in the NT1 samples by the two method, without enrichment and with HILIC enrichment

Supplementary Table S1: The relative abundance value of significant *N*-glycopeptides list obtained from NT1 and control samples.

Supplementary Figure S1: Unsupervised PCA for total identified glycopeptides in control and NT1 including each sample number

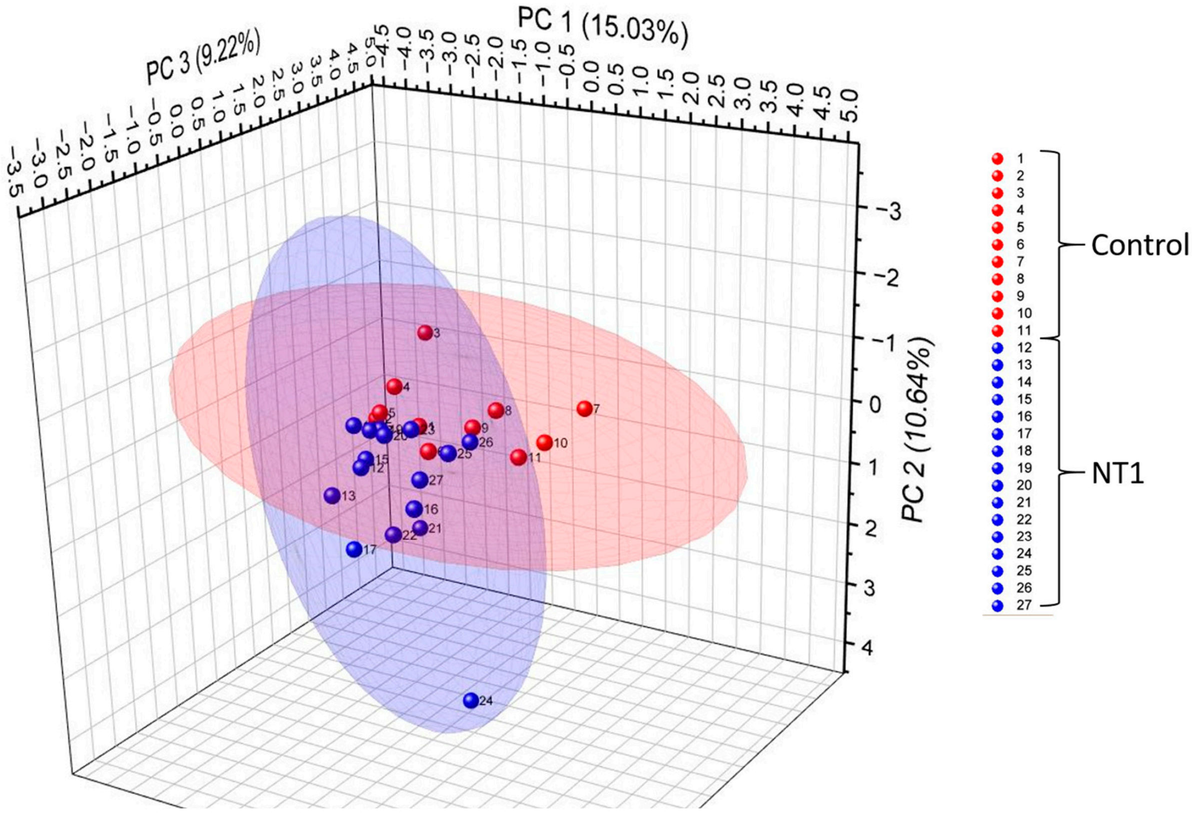

Supplementary Figure S2: Dot plots of the *N*-glycopeptides with statistically significant differences between control and NT1 samples

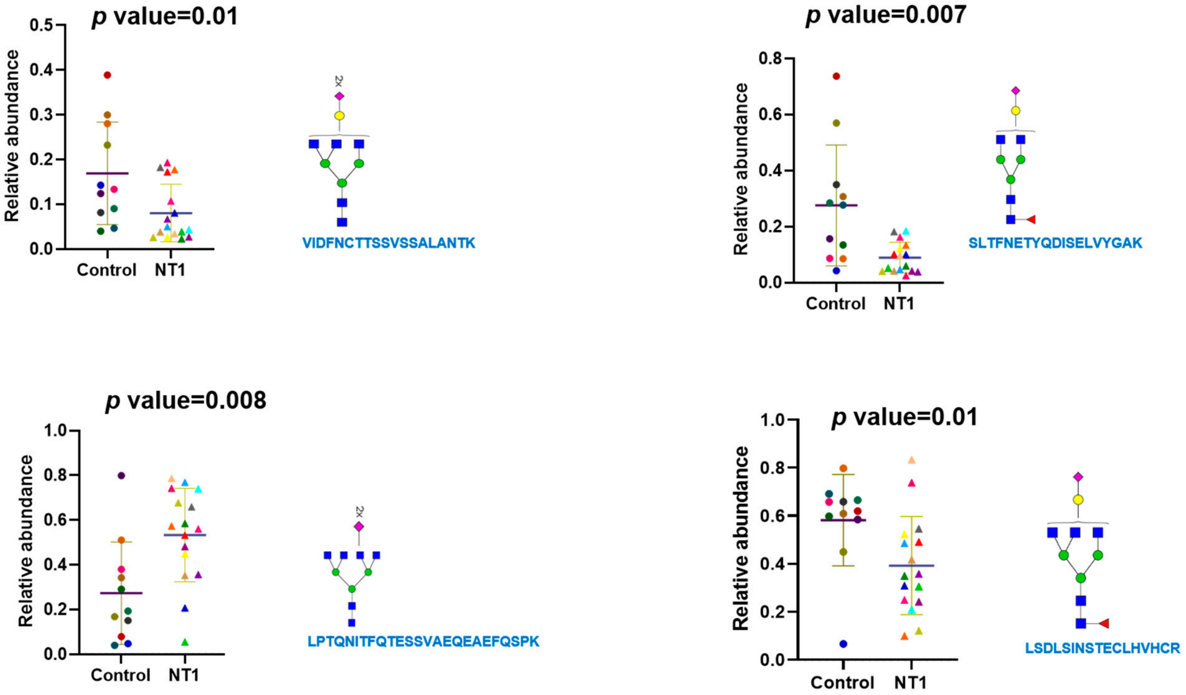

Supplementary Figure S3: Two significant isomers drived from control and NT1 blood serum samples for **A)** LDAPTNLQFVNETDSTVLVR +6-5-0-3, and B) FNLTETSEAEIHQSFQHLLR+7-6-0-4

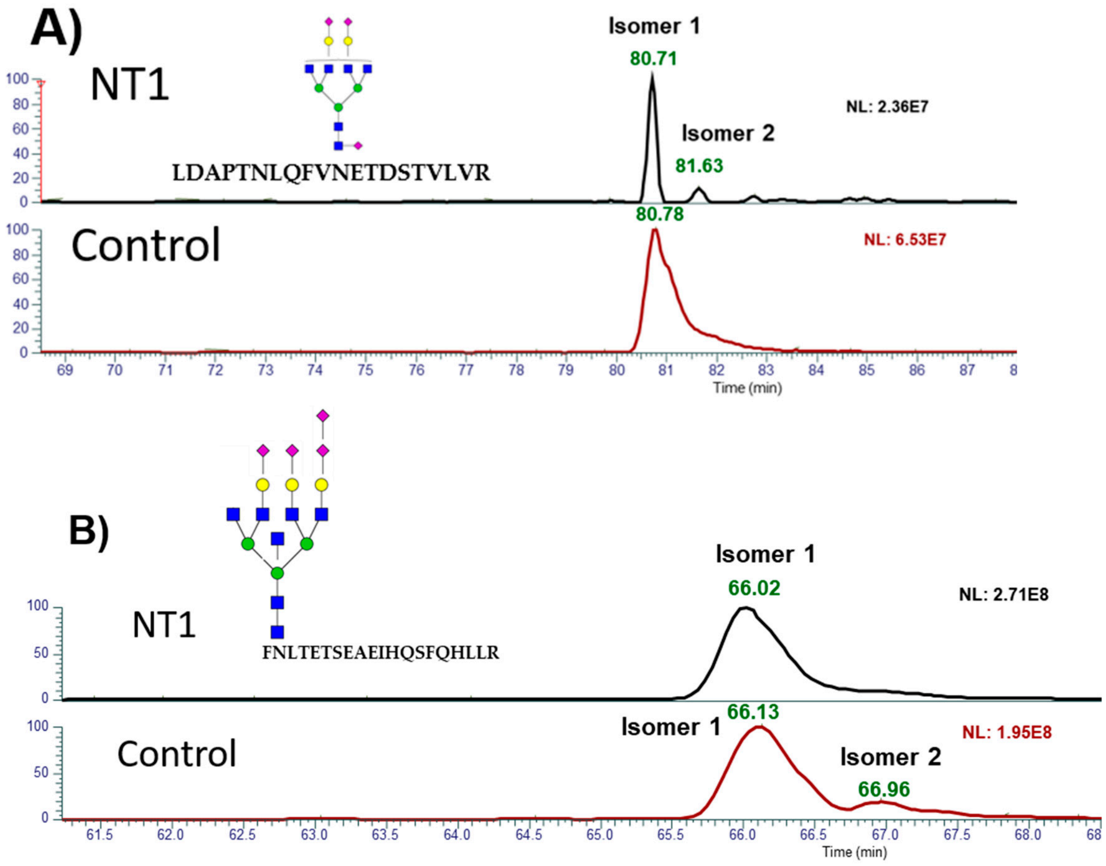

Supplementary Figure S4: Isomeric N-glycopeptides observed with the C18 (50cm) column **A)** sialylated glycopeptide with YPHKPEINSTTHPGADLQENFCR backbone, **B)** di-sialylated glycopeptide with LGACNDTLQQLMEVFK backbone, **C)** complex glycopeptide with NISDGFDGIPDNVDAALALPAHSYSGR, and **D)** di- sialylated glycopeptides with LQNNENNISCVER peptide backbone

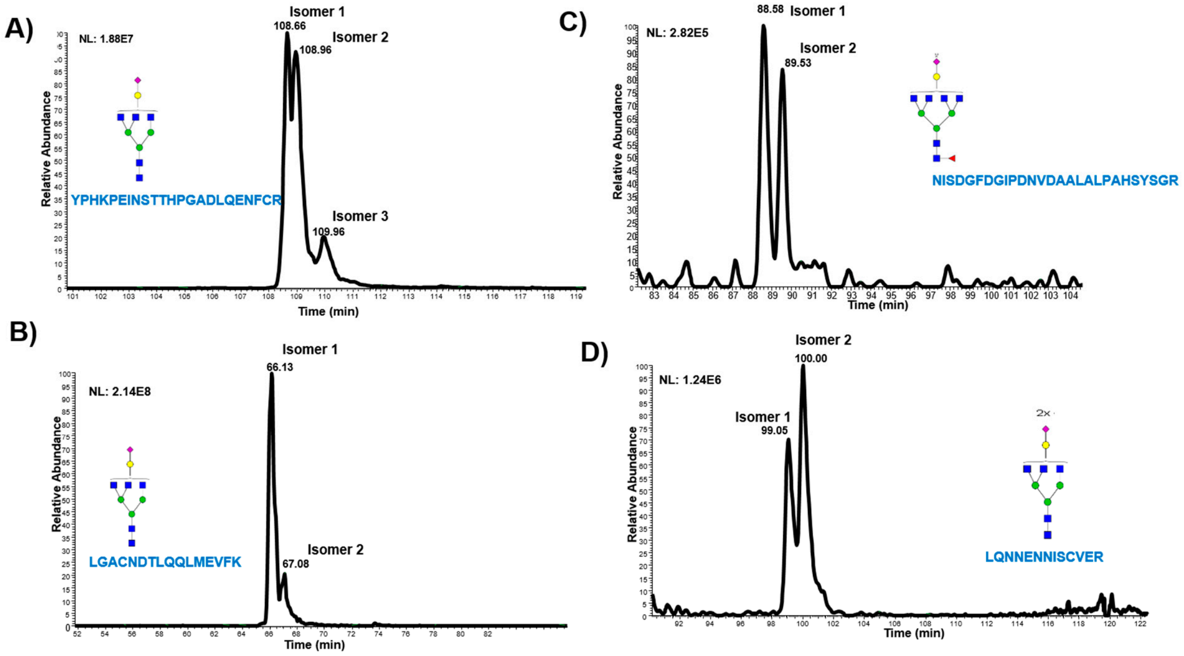

Supplementary Figure S5: A) Fragments of *N*-glycopeptides in NT1 including Y, B, and b/y ions in NT1 sample, **B)** glycan heterogeneity on the glycosylation site AALAAFNAQNNGSNFQLEEISR+5-4-0-2

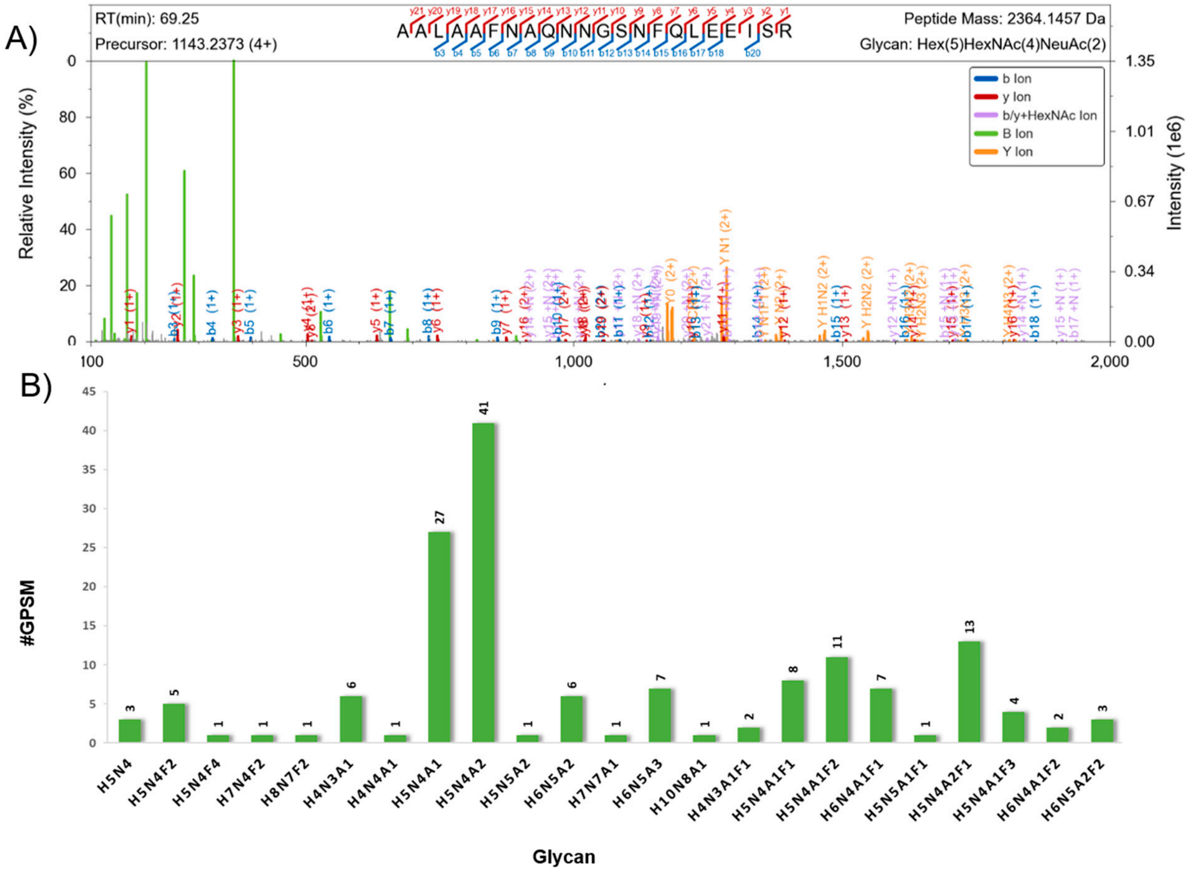

Supplementary Figure S6: MS spectra's from three technical replicates in NT1 samples for four *N*-glycopeptides to present reproducibility of enrichment method.

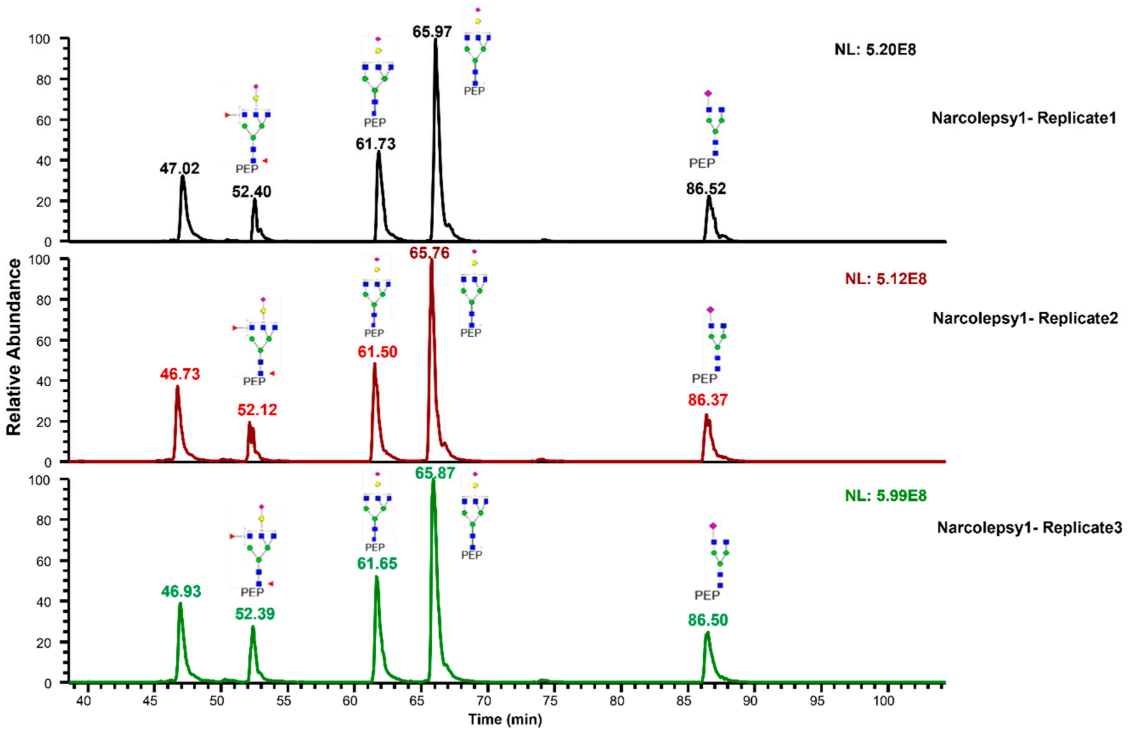

Supplementary Figure S7: Comparing number of different type of glycan in NT1 sample in two method, without enrichment and with HILIC enrichment

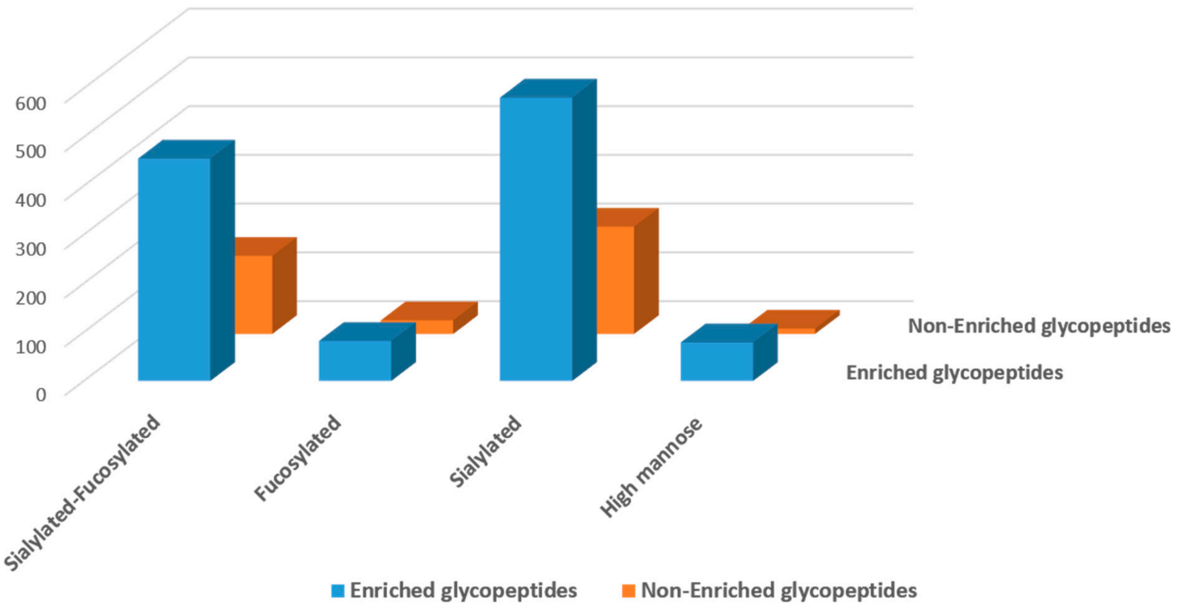

Supplementary Table S1: The relative abundance value of significant *N*-glycopeptides list obtained from NT1 and control samples.

Glycopeptide	CTRL-1	CTRL-2	CTRL-3	CTRL-4	CTRL-5	CTRL-6	CTRL-7	CTRL-8	CTRL-9	CTRL-10	CTRL-11	AUC Value
**ALPQPQNVTSLLGCTH Hex(5)HexNAc(4)NeuAc(1)**	0.03	0.07	0.10	0.04	0.06	0.02	0.06	0.14	0.03	0.19	0.14	0.80
**ENLTAPGSDSAVFFEQGTTR Hex(5)HexNAc(4)NeuAc(1)Fuc(1)**	0.02	0.001	0.0005	0.0001	0.00	0.01	0.01	0.00	0.003	0.001	0.00	0.75
**ENLTAPGSDSAVFFEQGTTR Hex(6)HexNAc(3)NeuAc(2)**	0.15	0.75	0.26	0.33	0.28	0.23	0.21	0.45	0.16	0.20	0.32	0.75
**FNLTETSEAEIHQSFQHLLR Hex(6)HexNAc(5)NeuAc(3)Fuc(1)**	0.24	0.85	0.90	0.59	0.44	0.29	0.57	0.53	0.13	0.18	0.37	0.78
**GDSGGPLVCMDANNVTYVWGVVSWGENCGKPEFPGVYTK Hex(5)HexNAc(4)NeuAc(2)**	0.003	0.001	0.0001	0.002	1.69E-5	0.00	0.00	0.001	0.001	0.001	0.00	0.75
**GFGVAIVGNYTAALPTEAALR Hex(5)HexNAc(4)NeuAc(1)**	0.21	0.47	0.01	0.06	0.18	0.18	0.18	0.42	0.10	0.13	0.12	0.74
**HGIQYFNNNTQHSSLFTLNEVK Hex(5)HexNAc(4)NeuAc(1)Fuc(2)**	0.03	0.01	0.04	0.02	0.03	0.12	0.04	0.04	0.14	0.28	0.09	0.81
**HGIQYFNNNTQHSSLFTLNEVKR Hex(6)HexNAc(5)NeuAc(2)**	0.11	0.09	0.24	0.07	0.09	0.07	0.24	0.13	0.06	0.23	0.25	0.77
**ICDLLVANNHFAHFFAPQNLTNMNK Hex(6)HexNAc(5)NeuAc(3)**	0.50	0.97	2.64	0.36	1.02	0.44	1.29	0.38	0.70	0.41	0.30	0.77
**IVLDPSGSMNIYLVLDGSDSIGASNFTGAK Hex(5)HexNAc(4)NeuAc(1)**	0.00	2.64E-6	0.01	0.004	0.01	0.00	0.004	0.04	0.05	0.04	0.00	0.80
**KEHETCLAPELYNGNYSTTQK Hex(5)HexNAc(4)NeuAc(1)**	0.26	3.47	0.05	0.12	0.003	0.10	0.01	0.26	1.58	0.04	0.17	0.76
**LEPVHLQLQCMSQEQLAQVAANATK Hex(5)HexNAc(4)Fuc(2)**	0.02	0.02	0.06	0.03	0.01	0.04	0.05	0.20	0.05	0.11	0.07	0.74
**LGNWSAMPSCK Hex(4)HexNAc(3)NeuAc(1)**	1.61	1.53	1.84	1.60	0.64	2.09	1.43	0.98	0.69	1.83	0.74	0.75
**LPTQNITFQTESSVAEQEAEFQSPK Hex(6)HexNAc(3)NeuAc(2)**	0.15	0.34	0.08	0.51	0.17	0.29	0.19	0.04	0.05	0.80	0.38	0.81
**LSDLSINSTECLHVHCR Hex(5)HexNAc(4)NeuAc(1)Fuc(1)F**	0.66	0.61	0.62	0.80	0.45	0.60	0.67	0.69	0.07	0.58	0.66	0.78
**NCGVNCSGDVFTALIGEIASPNYPK Hex(6)HexNAc(5)NeuAc(3)**	0.09	0.10	0.09	0.29	0.05	0.01	0.13	0.39	0.24	0.15	0.05	0.85
**NCGVNCSGDVFTALIGEIASPNYPKPYPENSR Hex(6)HexNAc(5)NeuAc(3)**	0.00	0.004	0.004	0.01	0.005	0.01	0.01	0.01	0.01	0.00	0.004	0.80
**NFTENDLLVR Hex(4)HexNAc(3)NeuAc(1)**	0.06	0.38	0.12	0.15	0.23	0.23	0.23	0.10	0.07	0.43	0.40	0.75
**NISDGFDGIPDNVDAALALPAHSYSGR Hex(5)HexNAc(4)NeuAc(2)Fuc(2)**	0.00	0.49	0.98	0.04	5.91	8.70	0.09	0.00	0.00	0.00	1.77E-6	0.75
**PLCVTLRCTNATVK Hex(3)HexNAc(7)**	0.11	0.24	0.13	0.22	0.07	0.15	0.53	0.30	0.48	0.22	0.24	0.85

**REGDHEFLEVPEAQEDVEATFPVHQPGNYSCSYR Hex(6)HexNAc(5)NeuAc(2)**	0.34	0.01	0.07	0.08	0.02	0.47	0.06	0.20	0.03	0.13	0.23	0.81
**RNPPMGGNVVIFDTVITNQEEPYQNHSGR Hex(6)HexNAc(5)NeuAc(1)Fuc(1)**	0.05	0.07	0.17	0.17	0.13	0.11	0.57	0.10	0.10	0.20	0.07	0.82
**SLTFNETYQDISELVYGAK Hex(4)HexNAc(4)NeuAc(1)Fuc(1)**	0.35	0.31	0.74	0.09	0.57	0.13	0.28	0.28	0.04	0.16	0.09	0.80
**SLTFNETYQDISELVYGAK Hex(6)HexNAc(4)NeuAc(1)Fuc(1)**	0.04	0.03	0.01	0.01	0.02	0.03	0.07	0.03	0.10	0.03	0.12	0.81
**SWPAVGNCSSALR Hex(6)HexNAc(5)NeuAc(1)**	0.09	0.06	0.22	0.03	0.10	0.09	0.10	0.21	0.11	0.16	0.06	0.74
**TMFPNLTDVR Hex(5)HexNAc(4)NeuAc(2)**	0.12	0.09	0.05	0.02	0.04	0.06	0.05	0.12	0.06	0.07	0.11	0.79
**VIDFNCTTSSVSSALANTK Hex(5)HexNAc(4)NeuAc(2)**	0.08	0.30	0.39	0.28	0.23	0.04	0.09	0.05	0.14	0.12	0.13	0.76
**VYIHPFHLVIHNESTCEQLAK Hex(5)HexNAc(4)NeuAc(2)**	0.03	0.05	0.02	0.02	0.03	0.02	0.11	0.05	0.16	0.07	0.06	0.78

Glycopeptide	NTI -1	NTI -2	NTI -3	NTI -4	NTI -5	NTI -6	NTI -7	NTI -8	NTI -9	NTI -10	NTI -11	NTI -12	NTI -13	NTI -14	NTI -15	NTI-16	AUC Value
**ALPQPQNVTSLLGCTH Hex(5)HexNAc(4)NeuAc(1)**	0.03	0.04	0.03	0.10	0.02	0.01	0.05	0.01	0.02	0.01	0.06	0.01	0.05	0.01	0.04	0.03	0.80
**ENLTAPGSDSAVFFEQGTTR Hex(5)HexNAc(4)NeuAc(1)Fuc(1)**	0.01	0.02	3.22 E-4	4.13 E-4	0.01	3.22 E-4	4.13 E-4	0.01	4.24 E-4	2.33 E-4	0.01	0.01	0.01	0.01	0.02	0.02	0.75
**ENLTAPGSDSAVFFEQGTTR Hex(6)HexNAc(3)NeuAc(2)**	0.41	0.40	0.82	0.84	0.30	0.74	0.29	0.40	0.28	0.68	0.39	0.27	0.82	0.18	0.45	0.22	0.75
**FNLTETSEAEIHQSFQHLLR Hex(6)HexNAc(5)NeuAc(3)Fuc(1)**	0.46	0.21	0.49	0.38	0.05	0.21	0.18	0.30	0.18	0.32	0.10	0.16	0.08	0.10	0.19	0.26	0.78
**GDSGGPLVCMDANNVTYVWGVVSW GENCGKPEFPGVYTK Hex(5)HexNAc(4)NeuAc(2)**	0.07	1.27 E-4	8.67 E-5	1.21 E-3	0.07	0.01	8.87 E-7	1.56 E-3	2.42 E-3	0.01	0.07	2.31 E-4	0.07	1.30 E-4	1.01 E-4	1.16E-4	0.75
**GFGVAIVGNYTAALPTEAALR Hex(5)HexNAc(4)NeuAc(1)**	0.73	0.44	4.60 E-3	0.19	3.02 E-3	0.00	0.88	3.02 E-3	0.06	0.01	0.00	0.01	0.10	0.04	1.53 E-3	1.53E-3	0.74
**HGIQYFNNNTQHSSLFTLNEVK Hex(5)HexNAc(4)NeuAc(1)Fuc(2)**	0.02	0.01	0.01	4.37 E-3	0.04	0.02	0.01	0.04	0.01	0.02	0.02	0.02	0.09	0.02	0.03	0.02	0.81
**HGIQYFNNNTQHSSLFTLNEVKR Hex(6)HexNAc(5)NeuAc(2)**	0.17	0.09	0.02	0.07	0.02	0.11	0.04	0.03	0.10	0.09	0.03	0.02	0.00	0.18	0.12	0.03	0.77
**ICDLLVANNHFAHFFAPQNLTNMNK Hex(6)HexNAc(5)NeuAc(3)**	0.35	0.06	0.43	0.61	0.08	0.24	0.29	0.59	0.52	0.09	0.07	0.21	0.55	0.18	0.58	0.50	0.77
**IVLDPSGSMNIYLVLDGSDSIGASNFT GAK Hex(5)HexNAc(4)NeuAc(1)**	0.07	0.04	0.01	0.13	4.31 E-3	0.05	0.01	0.03	0.09	0.04	0.01	0.01	4.37 E-3	0.08	0.11	0.02	0.80
**KEHETCLAPELYNGNYSTTQK Hex(5)HexNAc(4)NeuAc(1)**	0.67	4.74	1.01	6.47	0.42	3.45	4.03	0.77	0.33	0.55	0.53	0.04	0.23	0.02	0.15	2.01	0.76
**LEPVHLQLQCMSQEQLAQVAANATK Hex(5)HexNAc(4)Fuc(2)**	0.02	0.01	0.02	0.01	0.02	0.01	0.03	0.01	0.04	0.03	0.05	0.01	0.07	0.03	0.03	0.03	0.74
**LGNWSAMPSCK Hex(4)HexNAc(3)NeuAc(1)**	0.45	0.84	1.33	1.20	0.67	0.63	0.79	1.36	1.17	1.41	0.71	0.48	0.25	0.66	2.07	1.38	0.75
**LPTQNITFQTESSVAEQEAEFQSPK Hex(6)HexNAc(3)NeuAc(2)**	0.74	0.48	0.36	0.21	0.68	0.58	0.57	0.53	0.35	0.66	0.45	0.05	0.74	0.79	0.56	0.77	0.81
**LSDLSINSTECLHVHCR Hex(5)HexNAc(4)NeuAc(1)Fuc(1)F**	0.74	0.24	0.36	0.31	0.12	0.35	0.10	0.49	0.42	0.55	0.52	0.31	0.21	0.83	0.25	0.49	0.78
**NCGVNCSGDVFTALIGEIASPNYPK Hex(6)HexNAc(5)NeuAc(3)**	0.02	0.04	0.02	0.14	0.04	0.01	0.17	0.05	0.02	0.13	0.04	0.03	0.14	0.07	0.03	0.03	0.85
**NCGVNCSGDVFTALIGEIASPNYPKPY PENSR Hex(6)HexNAc(5)NeuAc(3)**	0.04	0.04	5.95 E-6	3.32 E-3	0.06	0.06	0.01	0.02	0.09	0.07	2.94 E-3	0.02	0.03	0.01	0.01	0.02	0.80
**NFTENDLLVR Hex(4)HexNAc(3)NeuAc(1)**	0.42	0.24	0.21	0.16	0.27	0.47	0.14	0.24	0.13	0.55	0.73	0.57	0.51	0.61	0.12	0.58	0.75
**NISDGFDGIPDNVDAALALPAHSYSGR Hex(5)HexNAc(4)NeuAc(2)Fuc(2)**	4.49	4.12	0.19	0.02	0.01	8.48	7.96	0.21	0.39	0.28	5.99	1.38	6.77	3.89	1.21	8.46	0.75
**PLCVTLRCTNATVK Hex(3)HexNAc(7)**	0.12	0.05	0.04	0.10	0.28	0.01	0.03	0.04	0.05	0.21	0.06	0.06	0.24	0.14	0.19	0.28	0.85

**REGDHEFLEVPEAQEDVEATFPVHQP GNYSCSYR Hex(6)HexNAc(5)NeuAc(2)**	0.02	0.01	0.01	0.03	0.05	0.03	0.01	0.01	0.01	0.06	0.04	0.05	0.01	0.04	0.04	0.03	0.81
**RNPPMGGNVVIFDTVITNQEEPYQNH SGR Hex(6)HexNAc(5)NeuAc(1)Fuc(1)**	0.05	0.06	0.06	0.07	0.10	0.07	0.06	0.03	0.11	0.09	0.07	0.06	0.07	0.09	0.10	0.11	0.82
**SLTFNETYQDISELVYGAK Hex(4)HexNAc(4)NeuAc(1)Fuc(1)**	0.03	0.04	0.04	0.10	0.04	0.06	0.13	0.10	0.04	0.18	0.12	0.05	0.18	0.10	0.16	0.05	0.80
**SLTFNETYQDISELVYGAK Hex(6)HexNAc(4)NeuAc(1)Fuc(1)**	0.12	0.34	0.06	0.10	0.05	0.17	0.20	0.03	0.03	0.24	0.04	0.05	0.71	0.07	0.06	0.10	0.81
**SWPAVGNCSSALR Hex(6)HexNAc(5)NeuAc(1)**	0.11	0.15	0.17	0.11	0.21	0.29	0.12	0.09	0.13	0.13	0.36	0.08	0.15	0.17	0.31	0.12	0.74
**TMFPNLTDVR Hex(5)HexNAc(4)NeuAc(2)**	0.02	0.02	0.01	0.02	0.04	0.01	0.03	0.03	0.04	0.02	0.08	0.08	0.04	0.14	0.04	0.03	0.79
**VIDFNCTTSSVSSALANTK Hex(5)HexNAc(4)NeuAc(2)**	0.19	0.03	0.07	0.08	0.03	0.02	0.18	0.17	0.04	0.18	0.03	0.04	0.04	0.03	0.11	0.05	0.76
**VYIHPFHLVIHNESTCEQLAK Hex(5)HexNAc(4)NeuAc(2)**	0.02	0.01	2.46 E-3	3.97 E-3	0.07	0.01	0.02	0.01	0.01	0.03	0.01	0.03	0.04	0.04	0.03	0.03	0.78
